# Meet the Fribbles: novel stimuli for use within behavioural research

**DOI:** 10.3389/fpsyg.2014.00103

**Published:** 2014-02-12

**Authors:** Tom J. Barry, James W. Griffith, Stephanie De Rossi, Dirk Hermans

**Affiliations:** ^1^Centre for the Psychology of Learning and Experimental Psychopathology, Faculty of Psychology and Educational Sciences, University of LeuvenLeuven, Belgium; ^2^Feinberg School of Medicine, Northwestern UniversityChicago, IL, USA

**Keywords:** attention, memory, anxiety, phobia, generalization, behaviour, avoidance, stimuli

## Abstract

Clinical researchers make use of experimental models for mental disorders. In many cases, these models use stimuli that are relevant to the disorder under scrutiny, which allows one to experimentally investigate the factors that contribute to the development of the disorder. For example, one might use spiders or spider-like stimuli in the study of specific phobia. More broadly, researchers often make use of real-world stimuli such as images of animals, geometrical shapes or emotional words. However, these stimuli are often limited in their experimental controllability and their applicability to the disorder in question. We present a novel set of animal-like stimuli, called Fribbles, for use within behavioural research. Fribbles have desirable properties for use in research because they are similar to real-world stimuli, but due to their novelty, participants will not have had previous experience with them. They also have known properties that can be experimentally manipulated. We present an investigation into similarity between Fribbles in order to illustrate their utility in research that relies on comparisons between similar stimuli. Fribbles offer both experimental control and generalisability to the real world, although some consideration must be made concerning the properties that influence similarity between Fribbles when selecting them along a dimension of similarity.

## INTRODUCTION

Investigations into the mechanisms that operate in the development, maintenance, and treatment of psychopathology benefit from the use of experimental models of clinically observed phenomena. It is important for experimental models to relate closely to the elements of psychopathology under investigation. An experimental model should allow for the manipulation of the same psychological processes that are evident in a mental disorder (Boddez et al., 2013), but relevant and valid experimental stimuli are essential for such experiments. 

The nature of stimuli is particularly relevant in experiments concerned with clinical phenomena where participants must respond to visual stimuli that are emotionally laden. Stimulus sets are generally situated somewhere along continuums of controllability as well as generalisability to the real world (external validity). Existing stimulus sets range from images of basic geometrical shapes (e.g., Vervliet et al., 2006) to pictures of real faces or houses (e.g., O’Toole et al., 2005) to animals presented on a computer screen (e.g., Vansteenwegen et al., 2005); from emotional words (e.g., as in the emotional Stroop; Williams et al., 1996) to sections of text depicting ambiguous, personally relevant scenarios in interpretation bias research (e.g., Mathews and Mackintosh, 2000) to virtual reality scenes containing complex environments (e.g., Baas, 2013). The position of each of these sets on the continuums of control and generalisaibility is dependent on the purpose of the experiment and the particular phenomena that is being studied.

Investigations into the generalization and treatment of fear, for example, may require that a stimulus set has as much controllability as possible. Controllability of a stimulus allows it to be systematically manipulated, which in turn facilitates the mapping of generalization gradients. For example, one can determine similarity from a prototype to all other stimuli within a set. The degree of similarity might then be correlated with the level of fear responding after an aversive conditioning event to a prototype (Vervliet et al., 2005). Geometrical shapes could provide such a purpose as they are easy to manipulate and so experimenters can quantify the degree of difference between stimuli in different experimental conditions. For example, if a small circle is paired with an electrocutaneous stimulus such that a participant responds fearfully to this circle in the absence of the shock, one can observe the generalization of fear to other circles of other similar sizes (Lissek et al., 2008). In this case, greater fear is associated with greater similarity to the original conditioned stimulus.

Shapes, however, may be inadequate for research concerned with translating its findings to pathological fear generalization, such as in specific phobias and other anxiety disorders. In specific phobias, fear might spread from an originally feared animal, such as a spider, to all other things with a similar appearance (e.g., other animals). Furthermore, in order for treatment of a spider phobia to succeed, new learning acquired through treatment must generalize to other experiences with spiders (Rowe and Craske, 1998). Research of this kind requires a stimulus set that has high generalisability to the kinds of things that people encounter – and can become afraid of – in their natural environment. Real-world stimuli such as images of animals or faces have been used to this effect (Rowe and Craske, 1998; Vansteenwegen et al., 2005). These stimuli are complex, which allows researchers to define similarity along several dimensions rather than along one dimension (e.g., size). They may however lack the controllability necessary to quantify similarity between stimuli for the purposes of reliably investigating generalization.

Even where control can be obtained – as in the use of facial morphs (Lenaert et al., 2012) – the use of real-world stimuli may be confounded by individual differences in the affective value of a particular image, as well as previous experience with particular kinds of stimuli. Such stimuli may only be of use in research evaluating behavioural responses in clinical or subclinical samples for whom one already expects a certain response, as with the presentation of spider images in a phobic population (e.g., Rowe and Craske, 1998). In a normal population such stimuli will have differential effects depending on individual learning histories.

**FIGURE 1:**
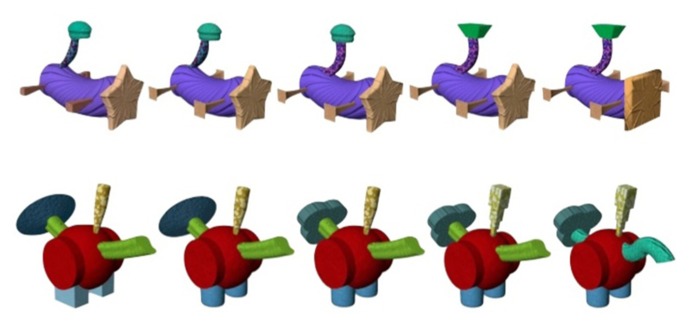
**Two Fribble species (FA1 and FC1).** From left to right, species prototypes to exemplars differing by one element; first differing in legs then tail1, tail2, and finally the head.

A stimulus set is needed with high controllability, high external validity, and minimal contamination from individual differences. Such a stimulus set must not be present in the real-world but must mimic the complexities of real stimuli whilst also having features that can be fully manipulated. A potentially fruitful set of stimuli that meets these demands are Fribbles. These are artificial, three-dimensional, combinations of shape, color, and texture that mimic the structures of real-world animals (Williams, 1998; see **Figure 1**). There are 12 species, each of which is constituted by a central body structure with four attached appendages. There are three unique body shapes – therefore the same body is repeated for two of the species, although the configuration of their appendages differs between these species. Within each species, there are a range of exemplars that differ from one another in terms of the number of appendage elements that they share with a prototype and in the characteristics of each appendage. Nevertheless, the central body and the location of appendages remain constant within a species.

Fribbles have four body parts that can vary and are thus the basis for manipulating similarity and difference including a head, legs, and two parts of the tail. Each appendage has three possible forms, the main difference between the variants being their shape, whereas the color and texture are intended to remain approximately similar. For example, the head of a Fribble could be a circle, a square, or a star. The specific colors and textures of the head only vary slightly. In the current database of Fribbles, the numerous possible combinations of appendages create eighty-one exemplars within each species. The positioning of appendages around the central body is such that Fribbles appear to be anatomically similar to real-world animals without actually appearing to represent any actual animal. The differences between species mirror differences found in the natural world. Thus, anatomical similarities are evident across Fribbles in the same way that rhinos differ from ducks whereas within-species changes in appendages such as the head mirror the same kinds of differences as might be evident between a python and a cobra for example (Williams, 1998). **Figure 1** illustrates these between and within species differences in two Fribble species.

The Fribbles offer a potentially valuable source of stimuli given their systematic within-species differences and anatomical structures like those of animals. This means they are both highly controllable and also relatable to things in the natural world without the confound of previous experience. Existing research using Fribbles is confined to the area of visual perception and categorization – both in terms of the fundamental cognitive processes involved (Williams, 1998; Casasanto, 2009; Dye and Ramscar, 2009) and the neuroscientific substrates underlying those processes (Barense et al., 2007; Behrmann and Williams, 2007; Hein et al., 2007). Fribbles were first used to show that people use stimulus features to assign category membership within and between related stimuli (Williams, 1998). Williams differed Fribbles within a species in terms of the number of shared features with a prototype. However, there was no investigation of whether, given the range of features on offer, all of the possible feature variants differed from the prototype features to the same degree. For example, if a prototype has a square head, a second Fribble that is the same in every way except it has a circular head will be judged as dissimilar to the prototype. However, it is unclear whether this second Fribble will be judged as equally dissimilar to the prototype as a third Fribble with a star-shaped head. We might also ask whether a fourth Fribble that has the same head as the prototype but has different legs will be judged as more or less dissimilar to the prototype as the second Fribble with the changed head. The question then becomes whether the number of shared features is sufficient to explain differences in similarity between Fribbles or whether research that uses Fribbles to form similarity comparisons needs to also consider the influence of different features and their forms on similarity. Knutson et al. (2012) considered these issues in their investigation into the contribution of the medial temporal lobe in detection of novel objects among similar objects. They used a modified Fribble set that differed from one another, within a species, in terms of the color of the bodies and the appendages in order to make these parts seem more or less salient. However, as Knutson et al. (2012) did not use the original Fribble set and they did not draw any conclusions regarding the relative contribution of the body parts of Fribbles to overall similarity between Fribbles, some investigation into these factors on similarity is still required in order to accurately select Fribbles for other research.

There has been no investigation into the degree of similarity between each of the possible variants of Fribble within each species. Until such an investigation takes place, the selection of individual stimuli from the wider Fribble set to form, or to avoid, any sort of similarity gradient is speculative and unsupported by data. In other behavioural research, the effect of such speculation is that individual features of a given stimulus set may have an unexpected confounding effect. For example, Lenaert et al. (2012) used facial morphs where a similarity gradient was derived from the gradual merging of two perceptually dissimilar faces (e.g., if Face A is 100% then the most similar face is 90% Face A and 10% of the perceptually dissimilar Face B). Face A was first paired with a lightning bolt image and in the subsequent phase they measured the extent to which participants expected the lightning bolt for each of the faces along the morphological gradient between Face A and B. They expected a linear decrease in expectancy as participants were shown faces further away from Face A and closer towards Face B. However, they reported a sigmoid “S-shaped” function where there was a sharp decline in expectancy in the middle of the gradient. This finding may be attributable to the saliency of the head hair in the images and the contribution of this to similarity judgements where the two faces at the middle of the gradient had noticeably different hair. This was despite their being positioned equally from the other faces on the morphological gradient. Lenaert et al. (2012) may have been able to control for or investigate the contribution of such features to the generalization of expectancy if an a priori investigation into similarity had taken place. Similarly, in their original presentation Williams (1998) suggests that they expect Fribbles to be linearly dissimilar from one another based on the number of shared features with a prototype, but they do not provide data on whether individual Fribble features can have different effects on similarity. Thus the present research attempts to provide data supporting the use of Fribbles in behavioural research with a focus on analysing the similarity between Fribbles to better inform their selection in future experiments.

We have two overarching hypotheses. First, we anticipate that Fribbles with increasing dissimilarity from the prototype, be it in terms of the number of common elements or the form that these elements take, will be judged as increasingly dissimilar by participants. Second, that the characteristics addressed within the first hypothesis have the same impact on similarity judgements between species.

Within the first hypothesis, we conducted separate analyses concerning each of the possible stimulus characteristics that might contribute towards similarity. First, we explored the influence of the number of common elements shared between an exemplar and the prototype on similarity judgements. Second, we explored whether body parts contribute equally to similarity, or whether some body parts have a greater impact on similarity than others; for example, whether changing the head of a Fribble has a greater impact on similarity to the prototype than if one was to change the legs. Third, we assessed the contribution of the different types within each body part to similarity judgements and whether this contribution is the same across all body parts and species. For example, are Fribbles with circular or star-shaped heads judged as being equally as similar to a prototype with a square head, and is this true for other body parts and their variants and does the same hold true for different species of Fribble.

## METHOD

### PARTICIPANTS

Participants were contacted through an e-mail database made up of participants from previous, unrelated, experiments and were asked if they would complete the survey. All participants reported having never seen or heard of the Fribbles. There was no reward for completing the survey. Seventy-six persons started the survey (51 females and 25 males), 41 of which completed all of the survey ratings (30 females and 11 males); that is, 41 participants had no missing data. The available data from all 76 persons were used in the analysis. Participants were mostly students at the University of Leuven and thus 82% of them were aged between 18 and 29 years.

### MATERIAL

Participants completed the survey using a web-based tool – SurveyMonkey®.

Four of the 12 Fribble species were used in the present investigation. Two of these species (referred to as FA2 and FC2) – of which eight exemplars were used – appeared within the training phase in order to give participants some experience as to the degrees of difference that would feature in the main investigation. These example comparisons included Fribbles for each number of possible common elements from the prototype (one to four). All exemplars from the other two species (FA1 and FC1) featured in the actual similarity survey. The Fribbles are designed and named such that in species FA1, a Fribble named 1211, for example, represents the same head, tail2 and legs as the prototype named 1111, but the two in the name represents a different type of tail1. Whereas, a Fribble in this species named 1121 denotes a change at tail2. The same is true for the head and legs of species FC1 but with 1211 referring to a change of tail2 and 1121 referring to a change of tail1. This difference in naming between species was due to the relative positioning of the tail parts on the Fribbles themselves so that tail1 always referred to the part attached directly to the body and tail2 the part with an intermediate connection between it and the body (see **Figure 1**). The Fribbles were in.jpeg image format and were of dimensions 319 × 240 mm.

### PROCEDURE

The survey was created in Dutch and English. Both versions were included in the same survey such that the English translation was presented in parenthesis after the Dutch version of each text. This method was used to accommodate participants at the University of Leuven, all of whom speak English and/or Dutch. Participants were informed that the survey would take approximately 15 min to complete and that their data would be held anonymously. A brief summary of the task was then presented, followed by demographic questions.

A single prototype Fribble was used within each species. During the survey, each trial compared the prototype Fribble for each species with an exemplar from the same species. Participants were asked to rate the similarity between the exemplar and the prototype on a scale from 1 (very dissimilar) to 20 (very similar). Each exemplar was presented one time along with the prototype Fribble from the same species. Stimuli remained on the screen until participants responded. The order of all presentations was randomized but the positioning of the prototype and exemplars remained constant throughout, with the prototype being presented on the left side of the screen and the exemplars on the right. The survey consisted of a training phase of 16 trials: eight trials with one species (FA2) and eight trials with another (FC2). When the training phase was complete, participants were instructed to click “next” in order to continue to the main survey, which consisted of 80 trials with one species (FA1) and 80 trials with another species (FC1). At the end of the experiment, participants were asked what strategy they used to make their similarity judgements.

## RESULTS

We used a type I error rate of α = 0.05 for all statistical tests. Huynh–Feldt corrected *p *values were used to account for violations of sphericity.

### ELEMENTS IN COMMON

**FIGURE 2:**
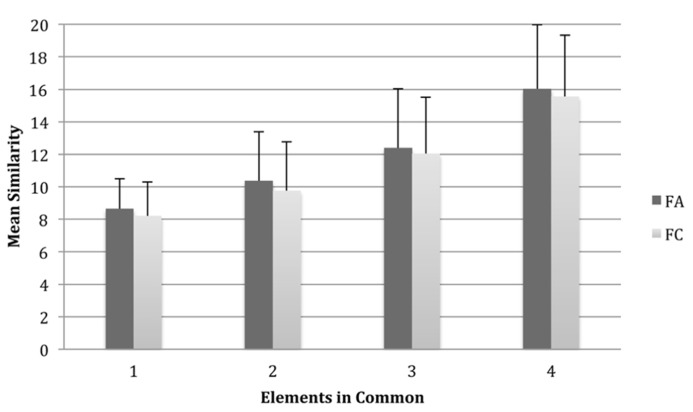
**Mean similarity ratings for exemplar Fribbles for each possible configuration of elements in common with the prototype Fribble for both species FA1 and FC1.** Similarity was scored on a scale ranging from 1 to 20 where “1” represented a highly dissimilar stimulus and “20” a highly similar stimulus. Higher means scores indicate greater similarity with the prototype. Error bars are one standard deviation.

In order to investigate the influence of the number of common elements shared between an exemplar and the prototype on similarity judgements, we performed a repeated-measures ANOVA with two within-subjects variables: elements in Common, (four levels; one, two, three, or four elements in common with the exemplar) and Species (two levels; FA1 and FC1) (see **Figure 2** for results). This showed a main effect of the number of common elements on similarity ratings and the eta squared (η^2^) value suggested that a large proportion of the variance in similarity ratings was explained by the number of common elements, *F*(3,123) = 150.12, *p* < 0.001, partial η^2^ = 0.79. Planned orthogonal polynomial contrasts showed a significant linear effect for these differences in similarity judgements, *F*(1,41) = 177.07, *p* < 0.001, and there was also a significant quadratic effect, *F*(1,41) = 37.01, *p* < 0.001. Fribbles with more elements in common with the prototype were judged to be more similar than those with fewer elements in common although this similarity gradient showed slight curvature suggesting that there was less difference between similarity ratings for three and four elements in common than for other comparisons. Further, those with four elements in common were judged much more similar than those with other numbers of common elements. There was also a main effect of species, *F*(1,41) = 5.38, *p* < 0.05, partial η^2^ = 0.12, wherein, overall, the FA1 exemplars were more often judged to be more similar to the prototype than the FC1 exemplars to their prototype, although the size of this effect was small. However, there was not a significant common elements × species interaction, *F*(3,123) = 0.28, *p* > 0.05, partial η^2^ < 0.01, which shows that the effect of common elements on similarity judgements did not differ significantly between species. When asked *post hoc* what strategy they used to make their judgements, participants most often reported using the number of common elements and the shape and form of the elements to make their judgements.

### BODY PART

Our second set of analyses explored whether body parts contribute equally to similarity, or whether some body parts have a greater impact on similarity than others. We computed indices of similarity, for each Fribble species separately, for each of the body parts using the following formula:

Similarity⁢ Index=a−[(b+c)/2]

In this formula, we use a to refer to the mean similarity score for Fribbles with the same body part type as the exemplar, *b* is the mean similarity score for Fribbles with the second type, and *c* is the mean score for the third variant. For example, where the prototype has a square head, *a *refers to Fribbles that also have the square head, *b *refers to Fribbles with a circular head and *c *refers to star-headed Fribbles. This was done for each of the body parts. Therefore, larger scores suggest that Fribbles with a matching body part type are rated more similar than Fribbles with a non-matching type.

A repeated measures ANOVA with two within-subjects variables, Body Part (four levels: head, tail1, tail2, legs) and Species (two levels: FA1 and FC1), showed a large main effect of Body Part on similarity, *F*(3,123) = 62.47, *p* < 0.001, partial η^2^ = 0.60. Interestingly, there was an interaction between Body Part and Species, *F*(3,123) = 25.93, *p* < 0.05, partial η^2^ = 0.39, suggesting that the effect of body parts on similarity differed between species.

**FIGURE 3:**
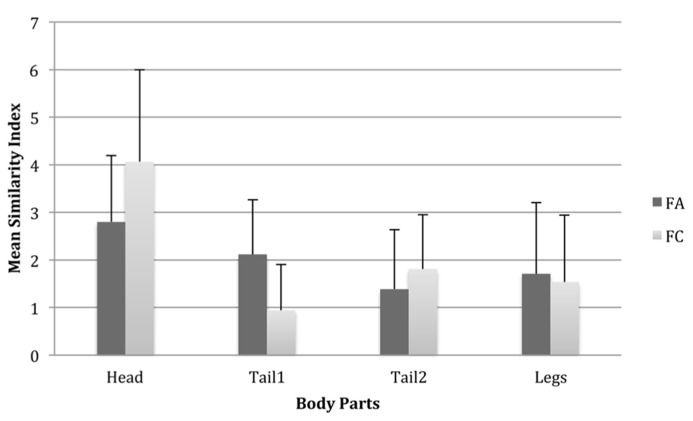
**Mean similarity index scores for each body part in both species, illustrating the effect of differing the type of each body part from the prototype.** Larger scores therefore indicate a larger effect of differing the type of a given body part from that of the prototype. Error bars are one standard deviation.

Fisher’s LSD tests showed that within the species, all body parts differed significantly from one another, with the head showing the greatest difference from all other body parts. This would suggest that having the head the same as the exemplar but all other parts different makes a Fribble more similar to the exemplar relative to if one of the other body parts remains the same as the exemplar. Results were very similar for the FC species with the exception that there was no significant difference between the legs and tail2 as to their contribution to similarity (see **Figure 3**). This suggests two things: first, body parts have a differential effect on similarity between species, and second that changes to body parts have equal effects on similarity with the exception that the head can have a larger impact on similarity ratings than the other body parts.

### BODY PART TYPE

#### Body part type between body parts

Next, we tested whether variations in body part type had differential impacts on similarity for different body parts. For example, whether an exemplar Fribble with a circular head when the prototype has a square head is more or less similar to its prototype than an exemplar Fribble with cylindrical legs when the prototype had rectangular legs. For this analysis we computed an index of difference formula as follows:

Difference⁢ index=|(b−c)|

Thus, the absolute value of the subtraction of *c* from *b*. Again, *b* refers to the mean similarity score for Fribbles with the second type body part and c the third type.

A repeated measures ANOVA with two within-subjects variables Body Part Type Difference (four levels: head, tail1, tail2, legs) and Species (two levels: FA1 and FC1) showed a significant main effect of different body part types on similarity, albeit with a small effect size, *F*(3,123) = 3.79, *p* < 0.05, partial η^2^ = 0.09, suggesting that the effect of differences in body part types on similarity ratings differed across the four body parts. However, there was a significant main effect of species, again with a small effect size, *F*(1,41) = 8.83, *p* < 0.01, partial η^2^ = 0.18, but the two-way interaction was not significant, *F*(3,123) = 2.49, *p* = 0.07, partial η^2^ = 0.06. As with the previous analyses, this confirms that there are likely to be between-species differences in the impact of changes to body parts and their types.

**FIGURE 4:**
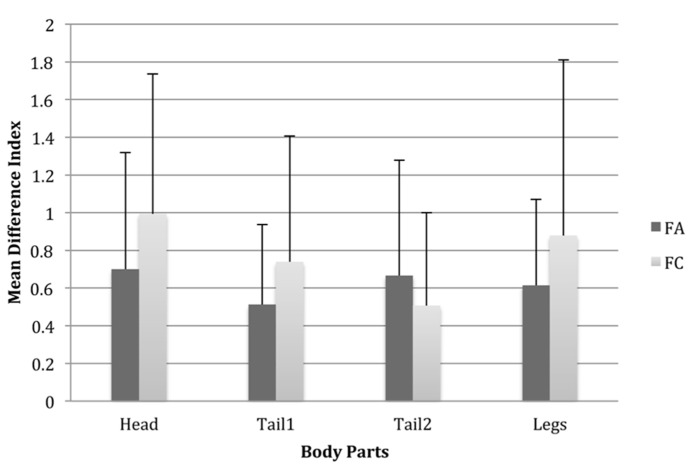
**Mean difference index scores for each body part for Fribbles in both species where the body part types differed from the prototype.** This was scored as the mean similarity score for Fribbles with the third part type subtracted from that of Fribbles with the second body part type. Error bars are one standard deviation.

Fisher’s LSD tests suggested that this main effect of Body Part Type is due mostly to significant differences in similarity between having the head different to the exemplar compared with having tail2 different, such that having a head of a different type makes a Fribble more dissimilar to the exemplar than having tail2 different from the exemplar (see **Figure 4**). This suggests that there are some inequalities among Fribble species as to the ways in which changes in the elements influence similarity ratings.

#### Body part type within body parts

Our final analysis explored, for each body part separately, whether each of the two alternative body part types were judged to be equally dissimilar from the prototype or whether one was judged to be more similar to the prototype than the other. A repeated measures ANOVA was performed on the mean similarity ratings for each body part type within each body part with two within-subjects variables – Body Part Type (three levels: type1, type2, and type3) and Species (two levels: FA1 and FC1). There were significant large to medium effects of Body Part Type on similarity for head, *F*(2,82) = 206.43, *p* < 0.001, partial η^2^ = 0.83; tail1, *F*(2,82) = 99.56, *p* < 0.001, partial η^2^ = 0.71; tail2, *F*(2,82) = 85.97, *p* < 0.001, partial η^2^ = 0.68; and legs, *F*(2,82) = 55.90, *p* < 0.001, partial η^2^ = 0.58. Interestingly, there were significant Body Part Type × Species interactions for head, *F*(2,82) = 19.26, *p* < 0.001, partial η^2^ = 0.32; tail1, *F*(2,82) = 45.62, *p* < 0.001, partial η^2^ = 0.53; tail2, *F*(2,82) = 6.13, *p* < 0.01, partial η^2^ = 0.13; and legs, *F*(2,82) = 15.43, *p* < 0.001, partial η^2^ = 0.27. This suggests that similarity differs as a product of the type of the body parts. Our comparisons also confirmed the expectation that having a body part type that is the same as the exemplar leads to judgements of greater similarity than if either of the other types are used; this is true for all body parts and both species that were analysed.

**FIGURE 5:**
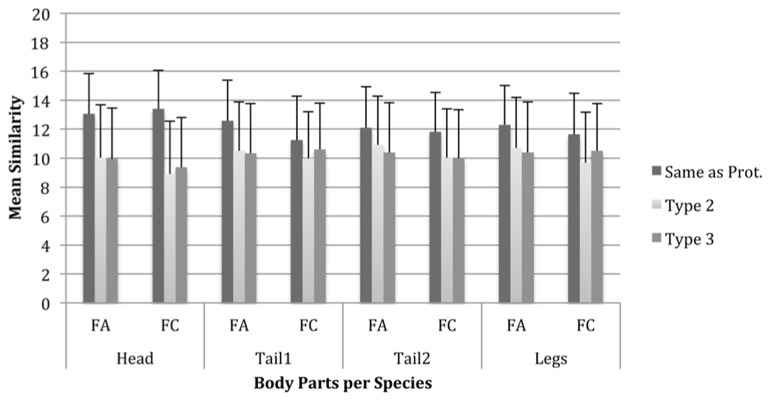
**Mean similarity ratings for exemplar Fribbles with each body part and per body part type and Fribble species.** Error bars are one standard deviation.

Planned comparisons for the FA1 species show that the main effect of head is due to significant differences in similarity between having the head the same and having either of the other types. This was also the case within the FC species (see **Figure 5**). Thus, an exemplar with the same head type is always rated as more similar to the prototype than exemplars with other head types. In the FA1 species, the same pattern of results is evident for the other body parts with the addition that for tail2 and the legs, there are also significant differences in similarity ratings between the body part types that are alternative to the exemplar within both tail2 and legs. In the FC species, there were also differences in similarity ratings between the alternative types for head, tail1and legs. For example, where the prototype has rectangular legs, exemplars with cylindrical legs may be judged to be significantly more similar to the prototype than exemplars with triangular legs. This suggests that alternative body part types may not be equal in the degree to which they differ from the prototype. However, this is only true for some body parts and this differs between species.

## DISCUSSION

The present investigation compared novel stimuli – Fribbles – in terms of their similarity to one another and the factors that contributed towards that similarity. This was done in order to illustrate the utility of the Fribbles for use within behavioural research, particularly generalization research, whilst also highlighting some important stimulus properties for consideration when selecting them.

First, as predicted, we replicated the previous findings from Williams (1998) that Fribbles that shared more elements with a given prototype within their species were judged to be more similar to that prototype than were Fribbles with fewer common elements. Second, overall having the same head as the prototype led to judgements of greater similarity than having any other body part as the same type as the prototype. Within this, we showed that the remaining body parts besides the head (e.g., tail1, tail2, and legs) all contributed similarly to judgements of similarity, with no body part – except for the head – showing any greater influence over similarity than any other. Finally, we also showed that some types of body part (e.g., a square head versus a circular head) differed in the extent to which they were judged to be similar to the prototype (e.g., a star-shaped head). Having the same body part type as the prototype for any body part was invariably judged to be more similar than having an alternative type. However, the two alternative variants may not be equal in the extent to which they differ from the prototype. For example, where the prototype head is a circle, the square head type may be viewed as more similar to this prototype than the star head type.

The finding that the heads of the Fribbles contribute more to judgements of similarity than any of the other features has important implications for the selection of Fribbles for behavioural research involving a dimension of similarity. This contradicts Williams (1998) original suggestion that similarity between Fribbles is based solely on the number of common elements. From a bottom-up perspective, the physical properties of the heads of the Fribbles (e.g., their color, size, or positioning) may be such that they are more salient to participants than the other body parts. It could also be due to a top-down perceptual preference for the heads similar to the suggestion that there is preferential facial processing in humans and non-human primates relative to the processing of other body parts (Pruce et al., 1996). These suggestions merit further investigation not only in terms of the Fribbles but also in terms of other similar stimulus sets where the head of the stimulus may contribute more to similarity judgements. In terms of selecting Fribbles to form a linear gradient of similarity, it may be necessary to make the other body parts more salient.

The results also suggested that some of the effects are not necessarily consistent across different species of Fribble. This means that different Fribble species may vary in the extent to which exemplars are judged to be similar to a given prototype. For example, a Fribble with two elements in common with its prototype might be judged to be more similar to the prototype than a Fribble from another species would be to its own prototype despite it also differing in the same two elements. It also means that, between species, different body parts and types may contribute differently towards similarity. These factors should be of consideration to researchers when selecting Fribbles for use within behavioural research. These findings add further detail to the findings of Williams (1998) and the original presentation of the Fribbles. It is clear that when selecting Fribbles for similarity-based research where comparisons are made between two separate species of Fribble as in the case of CS+ and CS- comparisons in conditioning research, one must consider the relative contribution of the possible variations in features to judgements of similarity. It is not sufficient to simply select Fribbles on the basis of the number of shared features. Future research could seek independent ratings to establish that their chosen Fribbles have features that do not differ significantly from one another in their contribution to similarity.

The value of the Fribbles in research is clear. They can provide affectively neutral control stimuli in research exploring cognitive and behavioural responses to emotional stimuli or stimuli within emotional contexts. For example, experiments with people with spider phobia might use the Fribbles as a neutral baseline from which to assess individual differences in approach and avoidance or the neural or physiological substrates of such behavioural responses (e.g., Ernst et al., 2012). This might be particularly useful where one is investigating the effects of approach or avoidance on attention towards stimulus features and one requires affectively neutral stimuli with numerous stimulus features (e.g., Förster et al., 2006). Real-world stimuli might not be suitable for such purposes given individual differences in valence. Also, shapes and words might lack the anatomical complexity and generalisability to clinical disorders necessary to provide an adequate comparison stimulus, particularly in neuroscientific paradigms.

Another potentially fruitful use for the Fribbles that is being pursued at present is in prospective investigations into the factors that contribute towards the development of clinical disorder. For example, after a conditioning experience where a Fribble is paired with an aversive event, one might relate the way that participants generalize their fear for this one Fribble to other, similar, Fribbles to the ways in which individuals differ in the ways they generalize their fears for real-world stimuli. Differences in this gradient of generalization might be attributable to such mechanisms as the forgetting of some of the Fribbles features and individual differences in memory specificity. This might mean that after conditioning to a Fribble with a square head, fear might generalize to all Fribbles with the square head irrespective of its other unique features (Riccio et al., 1992; Lenaert et al., 2012). This would be analogous to the way that after an aversive event such as a dog bite might lead to the development of a phobia when one begins to fear all dogs (Rowe and Craske, 1998).

Researchers could also use the Fribbles to explore the role of attention in anxiety disorders by measuring the orientation and duration of gaze towards Fribbles from within a species. One might expect individuals to be more likely to develop a phobia when they selectively attend to the “threatening” stimulus features in common with a Fribble paired with an aversive event relative to their gaze towards “safe” or unique features of other Fribbles within the same species (Bar-Haim et al., 2007; Armstrong and Olatunji, 2012).

Aside from the use of Fribbles, the methodological and statistical techniques employed herein could also be used to inform the selection of stimuli from other stimulus sets. For example, other research that tests generalization gradients amongst spiders of different kinds might use this procedure to quantify the degree of similarity between each spider. They could also use that data to compute similarity and difference indices and test the effects of changes in different stimulus features on similarity judgements between spiders and correlate this with the generalization of fear.

## LIMITATIONS

The online procedure in this study no doubt limits the conclusions that can be drawn. Given that participants completed the experiment on their own computer, we did not control for the computer or the screen that was used in the experiment. Differences in hardware between participants may have influenced the visual quality of the Fribbles that in turn may have influenced judgements of similarity. Nevertheless this study is valuable in simply presenting a potentially fruitful stimulus set and also in outlining the potential issues that must be considered when selecting them for research.

One limitation of this investigation was that there was no assessment of color-blindness or whether participants had normal or corrected-to-normal vision. Given the nature of the stimuli, accurate perception of features is essential in the ability to differentiate Fribbles from one another. Future research using the Fribbles should take this into consideration.

One other limitation of the current investigation could be that each Fribble was only compared to the prototype and not to other Fribbles within their species or any other species. This means that firm conclusions about the degree of difference between species cannot be made. However, this did not seem necessary given the apparent differences between species and the suggestion by the original creator, Williams (1998), that they were designed to differ between species in the same way that rhinos might be said to differ from ducks. Additionally, given the size of the Fribble stimulus set, it was beyond the scope of the present investigation to make all possible comparisons between all species and so only two Fribble species were compared. However, the recommendations made here in with regards to stimulus selection will no doubt transfer to all other Fribble species. Further research could confirm this by exploring similarity in the other Fribble species and potential differences in similarity between species. The use of a previously untested rating scale for the judgements of similarity might also be a limitation of the present study. A 20-point scale was used to attempt to capture the full variability in judgements of similarity between stimuli that are somewhat similar. Further research could confirm the reliability of this scale in assessments of similarity.

The number of trials in the experiment may also have been a limitation. Some participants reported modifying their strategy as the experiment progressed, relying more on a “feeling” than any conscious strategy as they began to experience fatigue. In these cases, participants may not notice some of the more subtle differences between body parts where they no longer pay attention to the individual features and instead look more generally at the Fribbles and form their judgements according to the composite.

## CONCLUSIONS

The Fribbles can be of great value to research, not only in terms of the experimental control they confer but also in terms of their applicability within the investigation of real-world clinical phenomena. This article has presented some of the possible ways in which Fribbles might be used in research – although this list is by no means exhaustive – and some of the factors that should be considered when selecting Fribbles.

## Conflict of Interest Statement

The authors declare that the research was conducted in the absence of any commercial or financial relationships that could be construed as a potential conflict of interest.

## References

[B1] Armstrong T. Olatunji B. O. (2012). Eye tracking of attention in the affective disorders: a meta-analytic review and synthesis. *Clin. Psychol. Rev.* 32 704–723 10.1016/j.cpr.2012.09.004 23059623 PMC3556338

[B2] Baas J. M. P. (2013). Individual differences in predicting aversive events and modulating contextual anxiety in a context and cue conditioning paradigm. *Biol. Psychol.* 92 17–25 10.1016/j.biopsycho.2012.02.001 22342768

[B3] Barense M. D. Gaffan D. Graham K. S. (2007). The human medial temporal lobe processes online representations of complex objects. *Neuropsychologia* 45 2963–2974 10.1016/j.neuropsychologia.2007.05.023 17658561

[B4] Bar-Haim Y. Lamy D. Pergamin L. Bakermans-Kranenburg M. J Van IJzendoorn M. H. (2007). Threat-related attentional bias in anxious and nonanxious individuals: a meta-analytic study. *Psychol. Bull.* 133 1–24 10.1037/0033-2909.133.1.1 17201568

[B5] Behrmann M. Williams P. (2007). Impairments in part-whole representations of objects in two cases of integrative visual agnosia. *Cogn. Neuropsychol.* 24 701–703 10.1080/02643290701672764 18066732

[B6] Boddez Y. Baeyens F. Luyten L. Vansteenwegen D. Hermans D. Beckers T. (2013). Validity of US expectancy. *J. Behav. Ther. Exp. Psychiatry* 44 201–206 10.1016/j.jbtep.2012.08.003 23207968

[B7] Casasanto D. (2009). Embodiment of abstract concepts: good and bad in right- and left-handers. *J. Exp. Psychol. Gen.* 138 351–367 10.1037/a0015854 19653795

[B8] Dye M. Ramscar M. (2009). “No representation without taxation: the costs and benefits of learning to conceptualize the environment,” in *Proceedings of the 31st Annual Conference of the Cognitive Science Society*, Amsterdam, The Netherlands. Available at:

[B9] Ernst L. H. Weidner A. Ehlis A. Fallgatter A. J. (2012). Controlled attention allocation mediates the relation between goal-oriented pursuit and approach – avoidance reactions to negative stimuli. *Biol. Psychol.* 91 312–320 10.1016/j.biopsycho.2012.08.004 22922017

[B10] Förster J. Friedman R. S. Özelsel A. Denzler M. (2006). Enactment of approach and avoidance behaviour influences the scope of perceptual and conceptual attention. *J. Exp. Social Psychol.* 42 133–146 10.1016/j.jesp.2005.02.004

[B11] Hein G. Doehrmann O. Müller N. G. Kaiser J. Muckli L. Naumer M. J. (2007). Object familiarity and semantic congruency modulate responses in cortical audiovisual integration areas. *J. Neurosci.* 27 7881–7887 10.1523/JNEUROSCI.1740-07.2007 17652579 PMC6672730

[B12] Knutson A. R. Hopkins R. O. Squire L. R. (2012). Visual discrimination performance, memory, and medial temporal lobe function. *Proc. Natl. Acad. Sci. U.S.A.* 109 13106–13111 10.1073/pnas.1208876109 22826243 PMC3420216

[B13] Lenaert B. Claes S. Raes F. Boddez Y. Joos E. Vervliet B. (2012). Generalization of conditioned responding: effects of autobiographical memory specificity. *J. Behav. Ther. Exp. Psychiatry,* 43 S60–S66 10.1016/j.jbtep.2010.12.010 21237446

[B14] Lissek S. Biggs A. L. Rabin S. J. Cornwell B. R. Alvarez R. P. Pine D. S. (2008). Generalization of conditioned fear-potentiated startle in humans: experimental validation and clinical relevance. *Behav. Res. Ther.* 46 678–687 10.1016/j.brat.2008.02.005 18394587 PMC2435484

[B15] Mathews A. Mackintosh B. (2000). Induced emotional interpretation bias and anxiety. *J. Abnorm. Psychol.* 109 602–615 10.1037//0021-843X.109.4.602 11195984

[B16] O’Toole A. J. Jiang F. Abdi H. Haxby J. V. (2005). Partially distributed representations of objects and faces in ventral temporal cortex. *J. Cogn. Neurosci.* 17 580–590 10.1162/0898929053467550 15829079

[B17] Pruce A. Allison T. Asgari M. Gore J. C. McCarthy G. (1996). Differential sensitivity of human visual cortex to faces, letterstrings, and textures: A functional magnetic resonance imaging study. *J. Neurosci.* 16 5205–5215 8756449 10.1523/JNEUROSCI.16-16-05205.1996 PMC6579313

[B18] Riccio D. C. Ackil J. Burch-Vernon A. (1992). Forgetting of stimulus attributes: methodological implications for assessing associative phenomena. *Psychol. Bull.* 112 433–445 10.1037/0033-2909.112.3.433 1438637

[B19] Rowe M. K. Craske M. G. (1998). Effects of varied-stimulus exposure training on fear reduction and return of fear. *Behav. Res. Ther.* 36 719–734 10.1016/S0005-7967(97)10017-1 9682527

[B20] Vansteenwegen D. Hermans D. Vervliet B. Francken G. Beckers T. Baeyens F. (2005). Return of fear in a human differential conditioning paradigm caused by a return to the original acquisition context. *Behav. Res. Ther.* 43 323–336 10.1016/j.brat.2004.01.001 15680929

[B21] Vervliet B. Vansteenwegen D. Baeyens F. Hermans D. Eelen P. (2005). Return of fear in a human differential conditioning paradigm caused by a stimulus change after extinction. *Behav. Res. Ther*. 43 357–371 10.1016/j.brat.2004.02.005 15680931

[B22] Vervliet B. Vansteenwegen D. Eelen P. (2006). Generalization gradients for acquisition and extinction in human contingency learning. *Exp. Psychol.* 53 132–142 10.1027/1618-3169.53.2.132 16909938

[B23] Williams J. M. G. Mathews A. MacLeod C. (1996). The emotional Stroop task and psychopathology. *Psychol. Bull.* 1 3–24 10.1037/0033-2909.120.1.3 8711015

[B24] Williams P. (1998). *Representational** Organization of Multiple Exemplars of Object Categories*. Available at: (accessed March 26, 2012)

